# Pancreatic B-13 Cell Trans-Differentiation to Hepatocytes Is Dependent on Epigenetic-Regulated Changes in Gene Expression

**DOI:** 10.1371/journal.pone.0150959

**Published:** 2016-03-08

**Authors:** Emma A. Fairhall, Michelle A. Charles, Philip M. E. Probert, Karen Wallace, Jennifer Gibb, Chandni Ravindan, Martin Soloman, Matthew C. Wright

**Affiliations:** 1 Institute of Cellular Medicine, Newcastle University, Newcastle, United Kingdom; 2 School of Medical Sciences, University of Aberdeen, Aberdeen, United Kingdom; Montana State University, UNITED STATES

## Abstract

The proliferative B-13 pancreatic cell line is unique in its ability to generate functional hepatocyte-like (B-13/H) cells in response to exposure to glucocorticoid. In these studies, quantitatively comparable hepatic levels of liver-specific and liver-enriched transcription factor and hepatocyte defining mRNA transcripts were expressed after 10–14 days continuous treatment with glucocorticoid. This conversion in phenotype was associated with increased Gr-α mRNA expression and translation of a functional N-terminally truncated variant protein that localized to the nucleus in B-13/H cells. A short (6 hours) pulse exposure to glucocorticoid was also sufficient to transiently activate the Gr and irreversibly drive near identical conversion to B-13/H cells. Examination of epigenetic-related mechanisms demonstrated that B-13 DNA was rapidly methylated and de-methylated over the initial 2 days in response to both continuous or pulse exposure with glucocorticoid. DNA methylation and glucocorticoid-dependent conversion to an hepatic B-13/H phenotype was blocked by the methylation inhibitor, 5-azacytidine. Conversion to an hepatic B-13/H phenotype was also blocked by histone deacetylase inhibitors. Previous experiments have identified N-terminal Sgk1 variant proteins as pivotal to the mechanism(s) associated with pancreatic–hepatic differentiation. Both continuous and pulse exposure to DEX was sufficient to result in a near-similar robust transcriptional increase in Sgk1c mRNA expression from undetectable levels in B-13 cells. Notably, expression of Sgk1c mRNA remained constitutive 14 days later; including after pulse exposure to glucocorticoid and this induction was inhibited by 5-azacytidine or by histone deacetylase inhibitors. These data therefore suggest that exposing B-13 cells to glucocorticoid results in a Gr-dependent pulse in DNA methylation and likely other epigenetic changes such as histone modifications that leads to constitutive expression of Sgk1c and irreversible reprogramming of B-13 cells into B-13/H cells. Understanding and application of these mechanism(s) may enhance the functionality of stem cell-derived hepatocytes generated *in vitro*.

## Introduction

The B-13 cell line is a progenitor cell line with an unusual capacity to generate comparatively near-functional hepatocyte-like (B-13/H) cells after treatment with glucocorticoid hormone alone [1–3]. This can be achieved on simple plastic culture sub-strata and appears to be related to a pathophysiological response of both rodent and human pancreas exocrine and/or progenitor cells to elevated glucocorticoid *in vivo* [4–6]. The capacity to generate near functional hepatocyte-like cells from B-13 cells is exemplified by the expression of functional drug metabolising enzymes in the B-13/H phenotype. B-13/H cells have been shown to express similar rat hepatocyte levels of total (carbon monoxide/reduced spectrally detectable) cytochromes P450 [7] and associated testosterone hydroxylation [2], para-nitrophenol hydroxylase activity [8], alkylresorufin dealkylase activities [9], Luciferin-IPA [9], paracetamol, diclofenac, bupropion and midazolam metabolism [7]. There is also limited expression of phase 2 conjugation activities such as glucuronidation and sulphation [10]. Given this apparent unique response, the B-13 cell line may have significant practical applicability in Toxicology as a platform to screen drug and chemical metabolism and toxicity [9,3]. However, the cell line is also a valuable model for understanding progenitor cell regulation in the pancreas and liver, since progenitors isolated from primary tissues are low in abundance, and on isolation, undergo a rapid irreversible transition to a rapidly proliferating mesenchymal phenotype that precludes their effective study [3].

Stem cells are a potential source of hepatocytes that could be used to study a variety of clinical and basic science questions and applications. However, at present, generating stem cell-derived hepatocytes remains challenging in terms of the resources required and level of functionality. At present, foetal-levels of expression of many genes preclude their use in many cases, most notably in toxicity studies [11]. This laboratory and others have therefore investigated a number of signaling pathways involved in B-13/H generation in part, to understand how stem cell-derived hepatic phenotype might be amplified to normal *in vivo* levels. In this respect, roles for the glucocorticoid receptor (Gr) [1], serine/threonine protein kinase 1 (Sgk1) [12], Wnt signalling [8] and induction of the liver-enriched transcription factors C/EBPα and C/EBPβ in the trans-differentiation have been identified [1,7].

In this paper, we demonstrate for the first time that glucocorticoid exposure results in the up-regulation of Gr mRNA expression and expression of a transcriptionally active N-terminally truncated Gr protein that shows increased nuclear localisation in B-13/H cells. We then demonstrate that Gr activation for just a short 6 hour period is sufficient to initiate trans-differentiation, that this is dependent on irreversible epigenetic changes at both the DNA and histone protein level and likely leads to the constitutive expression of a variant Sgk1c mRNA transcript.

## Materials and Methods

### Constructs

A sequence containing a concatamer of 4 glucocorticoid response elements (GREs) upstream of a eukaryotic minimal promoter was designed based on an alignment of the sequences of the GREs from the tyrosine amimontransferase gene (TAT); the tryptophan oxygenase gene (TO); the human metallothionein gene, hMT; the murine sarcoma virus (MSV), the human growth hormone gene (hGH) and the mouse mammary tumour virus (MMTV)–see S1 Fig. Oligonucleotide sequences were synthesized (plus and minus strand followed by annealing as previously outlined [13] and inserted into the pGL4.28 luciferase reporter plasmid construct (Promega, Southampton, UK) at the XhoI and BglII restriction sites using standard methods. Recombinants (GRE4–pGL4.28) were cloned and sequence checked prior to archiving the clone.

### Animal tissues and cells

Male Sprague-Dawley rats (230-250g body weight) were purchased from Charles River (Kent, UK) and housed in accordance with Home Office guidelines. Rat hepatocytes were isolated by collagenase perfusion as outlined [9] under a UK Home Office license with Newcastle University Local Ethics Committee approval that includes specific approval for this study. Rat liver and pancreas tissue was isolated during perfusion procedures to reduce the number of animals used and total RNA isolated from snap frozen tissue or tissue homogenates prepared as outlined [2].

### B-13 Cell Culture

B-13 cell line was sub-cloned from the rat adenocarcinoma AR42J cell line (archived at the American Type Culture Collection and available from a variety of commercial suppliers) by the Kojima lab [14]. For an extensive review of the origin of both the AR42J and B-13 cell lines, see [3]. B-13 cells were routinely cultured in 6 well plates in low glucose Dulbecco’s minimum essential medium (DMEM) supplemented with 10% (v/v) foetal calf serum (FCS), 80u/ml penicillin and 80μg/ml streptomycin (1.5 mls/well). Cells were incubated at 37°C in a humidified incubator gassed with 5% CO_2_ in air. Dexamethasone (DEX) was purchased from the Sigma Chem Co. (Poole, UK) and was added to medium from 1000-fold concentrated ethanol vehicle solvated stocks, control cells were treated with 0.1% (v/v) ethanol alone as control. Medium was routinely replaced every 2–3 days, unless otherwise stated. Cells treated continuously for 10–14 days with DEX are referred to as B-13/H cells (B-13 cells with an hepatocyte phenotype). For all DEX time course experiments, cells were seeded 24 hours prior to DEX administration. For cells treated with 10 nM DEX for 6 hours only, after six hours exposure to DEX, cells were washed three times with 3mls/well of PBS (137mM NaCl, 2.7mM KCl, 10mM phosphate pH 7.4) and media replaced with standard DMEM culture media (i.e. supplemented with 10% (v/v) FCS, 80u/ml penicillin and 80μg/ml streptomycin). Typically 85% - 95% of B-13 cells trans-differentiated into B-13/H cells with continuous DEX treatment as judged by phenotypic observation [4]. B-13/H cells do not proliferate and therefore to reduce interference from a minor proliferating population of B-13 cells in analyses, B-13 cells were exposed to DEX once they had achieved a near confluence (reducing the expansion of B-13 cells). Maximal trans-differentiation occurred after 10–14 days treatment with further treatment not increasing the number of trans-differentiated cells/well.

### RT-PCR and qRT-PCR

Total RNA was purified using Trizol (Invitrogen, Paisley, UK). RT-PCR was carried out as previously outlined and using primer sequences given in [9]. Quantitative RT-PCR employed the use of an Applied Biosystems7500 Real-Time PCR machine, using SYBR® Green Jumpstart™ master mix (Sigma-Aldrich, UK), with 2μM forward and reverse primer. Additional primer sequences and primers for RT-PCR are given in Table 1.

**Table 1:** Primers used for RT-PCR and qRT-PCR.

Oligo ID		Primer sequence (5’-3’)	Comments
**RT-PCR**			
**rSgk1c**	US	AAGTAACCCCAGCCTTCACC	Will amplify rat Sgk1c - also referred to as variant 2 (NM_001193569.1) cDNA sequence of 143bp–see Wallace et al., 2011 supplementary data section.
	DS	GCATGCATAGGAGTTGTTGG	
**rGr-α**	US	GCGACAGAAGCAGTTGAGTCATC	Will hybridise to rat Gr cDNA (NM_012576.2) sequence of 103bp. Also transcript variants X1 (XM_008772065.1) and X2 (XM_008772066.1).
	DS	CCATGCCTCCACGTAACTGTTAG	
**rGr-β**	US	GCGCTTGAGGCTAAGATAGCT	Will hybridise to rat Gr-β cDNA sequence of 108bp as reported by DuBois et al., (2013).
	DS	CCCATGTTTCTGCCTCTTTCTTTG	
**rDnmt1**	US	TACGCAAGGTTTGAGTCCCC	Will hybridise to rat Dnmt1 cDNA (NM_053354.3) sequence of 191bp.
	DS	CCCAGTCGGTAGACAACACC	
**rDnmt3a**	US	GCTATTTACAGAGCTTCGGGC	Will hybridise to rat Dnmt3a cDNA transcript variant 2 (NM_001003957.1) sequence of 125bp.
	DS	TGGCTTTCCTCCACAGCATT	
**rDnmt3b**	US	CCAAGGCGTATTCGTCGC	Will hybridise to rat Dnmt3b cDNA (NM_001003959.1) sequence of 161bp.
	DS	TACGTTTACTTGGGCCGCTT	
**rTet1**	US	TATATGGCTGTGCTGCCCAA	Will hybridise to rat Tet1 variant X1cDNA (XM_006256398.2) sequence of 212bp.
	DS	CGATGGGCCATTGCTTGATG	
**rTet2**	US	TGTTGTCAGGGTGAGAATCCAG	Will hybridise to rat Tet2 cDNA variant X1 (XM_006233347.2), X2 (XM_008761534.1) and X3 (XM_227694.7) sequences of 225bp.
	DS	CCTGTAGGCATCAGGTGCAA	
**rTet3**	US	CCCTTGCCTGAAGCATCTCA	Will hybridise to rat Tet3 cDNA (XM_008763095.1 and several variants) sequence of 311bp.
	DS	GCCGAGGTACCATTCCCAAA	
**rTgd**	US	GCTTCAACCGCACAAGATCC	Will hybridise to rat Tgd cDNA (NM_053729.1) sequence of 196bp.
	DS	CTGCAGGTCGAACGTGTACT	
**rMecp2**	US	GCGACGTTCCATCATTCGTG	Will hybridise to rat Mecp2 cDNA (NM_022673.2) sequence of 77bp.
	DS	TAAGCTTTCGCGTCCAACCT	
**rAicda**	US	AAATGGCCCCACGTTCCTAC	Will hybridise to rat Aicda cDNA (NM_001100779.1) sequence of 428bp.
	DS	TGGTCTTGTGCCTTGCCTTT	
**Gapdh**	US	TGACATCAAGAAGGTGGTGAAG	Will amplify rat (NM 017008), human (NM 002046) or mouse (NM 008084) glyceraldehyde 3 phosphate dehydrogenase cDNA sequence of 243bp.
	DS	TCTTACTCCTTGGAGGCCATGT	
**qRT-PCR**			
**rAmylase**	US	TGGCCGCGTGACAGAATTCAAGTA	Will hybridise to rat pancreatic Amy2a3 cDNA (NM_031502.1) sequence of 175bp at 59^°^C to produce a single amplimer.
	DS	TCCAGCACCATGTCCTCGCT	
**rCebp-α**	US	ATAAAGCCAAACAGCGCAAC	Will hybridise to rat Cebp-α cDNA (NM_012524.2) sequence of 67bp at 54^°^C to produce a single amplimer.
	DS	CGGTCATTGTCACTGGTCAA	
**rCebp-β**	US	CTTCAGCCCCTACCTGGAG	Will hybridise to rat Cebp-β cDNA (NM_024125.4) sequence of 88bp at 55^°^C to produce a single amplimer.
	DS	GAGGTCGGAAAGGAAGTCGT	
**rCyp2e1**	US	CTGACTGTCTCCTCATAGAGATGG	Will hybridise to rat xCyp2e1 cDNA (NM_031543.1) sequence of 78bp at 55^°^C to produce a single amplimer.
	DS	TCACAGAAACATTTTCCATTGTGT	
**rAlbumin**	US	TGGTCGCAGCTGTCCGTCAGA	Will hybridise to rat albumin cDNA (NM_134326.2) sequence of 192bp at 61^°^C to produce a single amplimer.
	DS	CGCATTCCAACAGGTCGCCG	
**rCpsI**	US	TGTGAAGGTCTTGGGAACATCGGT	Will hybridise to rat CpsI cDNA (NM_017072.1) sequence of 173bp at 59^°^C to produce a single amplimer.
	DS	ACCGAATCATCACAGGGTAGCCAA	
**rHNF1-α**	US	CTCAGCACCAGTCCCACAG	Will hybridise to rat Hnf1-α cDNA (NM_012669.1) sequence of 64bp at 56^°^C to produce a single amplimer.
	DS	CGTTGGAGTCAGAACTCTGGT	
**rHnf1-β**	US	TCCTCTCACCTGACAGTAAAATGAT	Will hybridise to rat Hnf1-β cDNA (NM_013103.1) sequence of 73bp at 55^°^C to produce a single amplimer.
	DS	ATATTCGTCAAGGTGCTGACC	
**rHnf4-α**	US	AGCAATGGACAGATGTGTGAGT	Will hybridise to rat Hnf4-α cDNAs from transcript variant 2 (NM_001270931.10 and Hnf4α transcript variant 1 (NM_022180.2) sequence of 93bp at 55^°^C to produce a single amplimer.
	DS	AGATCCAGAGCCACTTGGTG	
**rFoxa1**	US	CATGAGAGCAACGACTGGAA	Will hybridise to rat Foxa1 cDNA (NM_012742.1) sequence of 83bp at 54^°^C to produce a single amplimer.
	DS	CCGGAGTTCATGTTGCTGA	
**rFoxa2**	US	TCCCTGGGACTTAACTGTAACG	Will hybridise to rat Foxa2 cDNA (NM_012743.1) sequence of 74bp at 55^°^C to produce a single amplimer.
	DS	ACGGCTCCCAGCATACTTT	
**rFoxa3**	US	ACTACCGGGAGAACCAGCA	Will hybridise to rat Foxa3 cDNA (NM_017077.2) sequence of 61bp at 57^°^C to produce a single amplimer.
	DS	TCATTGAAGGACAGCGAGTG	
**rGr-α**	US	GTGCTGACATGTGGAAGCTG	Will hybridise to rat Gr-α cDNA (NM_012576.2) sequence of 86bp at 54^°^C to produce a single amplimer. Since this primer pair amplifies a region of the Gr transcript encoded from exons 1 and 2, this primer pair will also amplify any Gr-β transcripts.
	DS	CAATCGTTTCTTCCAGCACA	
**rMecp2**	US	TGGTAGCTGGGATGTTAGG	Will hybridise to rat Mecp2 cDNA (NM_022673.2) sequence of 219bp at 55^°^C to produce a single amplimer.
	DS	AACTTCAGGGGTTTCTCTTTGAG	
**18s rRNA**	US	CCCGAAGCGTTTACTTTGAA	Will amplify 136bp of rat 18s rRNA.
	DS	CCCTCTTAATCATGGCCTCA	

### Western blotting

Western blotting was performed essentially as previously outlined [4]. Antibodies for Cyp2e, Cps1, Cebpα/β, Oct4, Sox, c-myc, Klf4 and Gr (BuGR2) were purchased from Abcam (Cambridge, UK), β-actin from Sigma (Poole, Dorset) and albumin from MP Biomedicals (Cambridge, UK). Anti-amylase antibody was purchased from Santa Cruz Biotechnology (Santa Cruz, CA). Detection was achieved using appropriate horseradish-peroxidase-conjugated anti-IgG secondary antibodies and chemiluminescence (ECL, GE Healthcare, Amersham, UK).

### Immunostaining

B-13 cells were grown on chamber slides and permeabilised with ice-cooled methanol for 10 minutes before washing in PBS and fixing in 2% w/v formaldehyde, 0.2% gluteradehyde in PBS. Non-specific binding of antibodies was blocked through incubation with 20% (v/v) bovine foetal calf serum (FCS) in PBS for 20 minutes at room temperature. Samples were then incubated with the relevant primary antibody diluted in 0.05% (v/v) FCS and left to incubate overnight at 4°C. Samples were washed twice in PBS before applying appropriate FITC conjugated secondary antibodies for 1 hour at RT. Cells were mounted in Vectashield with DAPI (Vector Labs) prior to examination.

### Transfection

B-13 cells were seeded into 6 well plates 24 hours prior to transfection and cultured in standard culture media. Cells were transfected with 2 μg GRE4–pGL4.28 reporter plasmid (or pGL4.28, see S1 Fig) and 0.2 μg Renilla Luciferase (RL-TK) per well using Lipofectamine® transfection reagent, according to the manufacturer’s instructions, and cultured for 48 hours prior to treatment. Luciferase activities were analysed using a Dual Glo® luciferase assay system (Promega, Southampton, UK) and a luminometer. Luciferase activities were normalised to renilla activities, to control for any variations in transfection efficiencies between wells.

### DNA methylation

Total global methylation was determined using a Sigma Imprint® Methylated DNA quantification kit, essentially according to the manufacturer’s instructions. Typically, 100ng of DNA from each treatment was analysed in compared to standard methylated DNA provide in the kit. Relative global methylation levels were calculated as percentage compared to the fully methylated control.

### Statistical analysis

The student's T test (two tailed) was used to test for statistical significances.

## Results

### B-13 differentiation to B-13/H cells involves a co-ordinated alteration in gene expression leading to hepatic levels of hepatocyte-specific/hepatocyte-enriched gene expression

**Fig 1:**
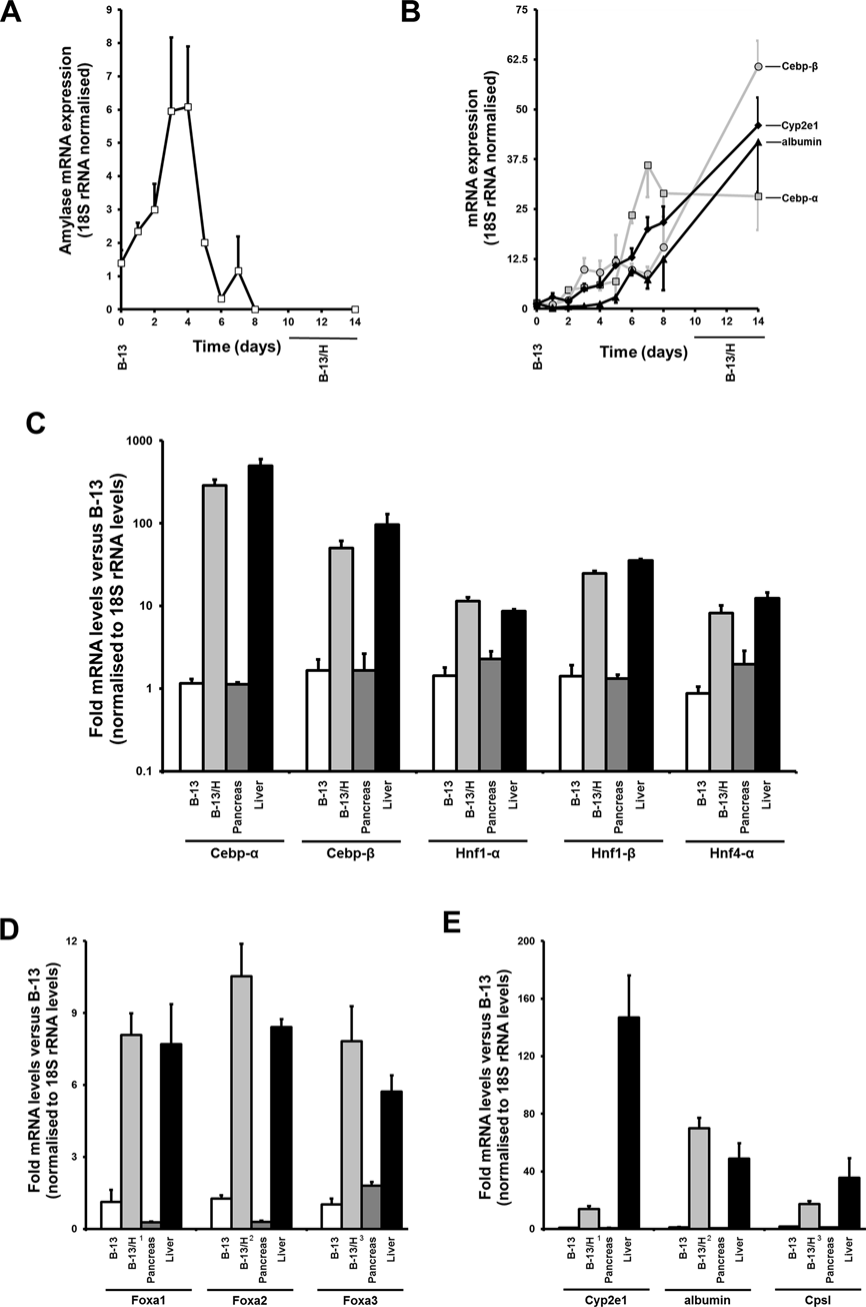
Quantitative RT-PCR determination of transcripts in B-13, B-13/H cells and comparison to rat liver and pancreas levels. Time course for the expression of amylase mRNA (**A**) or Cebp-α (-■-), Cebp- β (-●-), albumin (-▲-) and Cyp2e1 (-♦) mRNAs (**B**) in B-13 cells after treatment with 10nM DEX. Data are mean and standard deviation of three independent experiments. B-13 cells treated with 0.1% (v/v) ethanol (i.e. vehicle control) had minimal effect on all transcripts and are not shown for clarity. After 3–4 days, B-13 cells unexposed to DEX required sub-culturing since they continued to proliferate to avoid cell death. Therefore, they were not appropriate controls for B-13 cells treated with DEX after this time since DEX treatment resulted in a cessation of proliferation and sub-culture was not required. **C—E,** Expression levels of the indicated 18S rRNA-normalised transcript, relative to the levels present in B-13 cells, data are the mean and standard deviation of at least 3 separate cell samples/rat tissue samples.

The B-13 cell line was derived from the exocrine rat pancreas (for review, see [3]) and Fig 1A demonstrates that treatment with 10nM dexamethasone (DEX) glucocorticoid resulted in an initial increase in the expression of pancreatic amylase mRNA as previously reported [1]. Fig 1B shows that Cebp-α expression was induced early (typically around day 2), followed by Cebp-β. After day 5 there was a more robust induction of the Cebp mRNA transcripts and induction in the expression of liver-specific (or enriched) mRNAs (CpsI, Cyp2e1 and albumin). This latter change occurred around the time that amylase expression was markedly suppressed (between 4–6 days after DEX treatment–Fig 1A). The notable feature of the B-13 response was the quantitative change in expression of liver-specific and liver-enriched transcripts suggestive of a robust coordinated alteration to an hepatic phenotype, despite the simplicity of the culture conditions. To test this conclusion, the quantitative expression of liver-specific/enriched mRNAs was examined for the first time by direct quantitative comparison to intact rat liver and rat pancreas tissue. Fig 1C and 1D demonstrate comparable mRNA levels of expression of Cebp, Hnf and Foxa transcription factors in liver and B-13/H cells, and comparable levels of expression of functional hepatic genes (Fig 1E).

Glucocorticoid exposure therefore stimulated B-13 cell trans-differentiation to B-13/H cells which, based on quantitative levels of expression of liver-specific and liver-enriched transcription factor and hepatocyte defining mRNA transcripts, demonstrated a similar phenotype to hepatocytes.

### Glucocorticoid exposure results in an induction of Gr mRNA expression and expression of a transcriptionally active N-terminally truncated Gr protein with increased nuclear localisation in B-13/H cells

**Fig 2:**
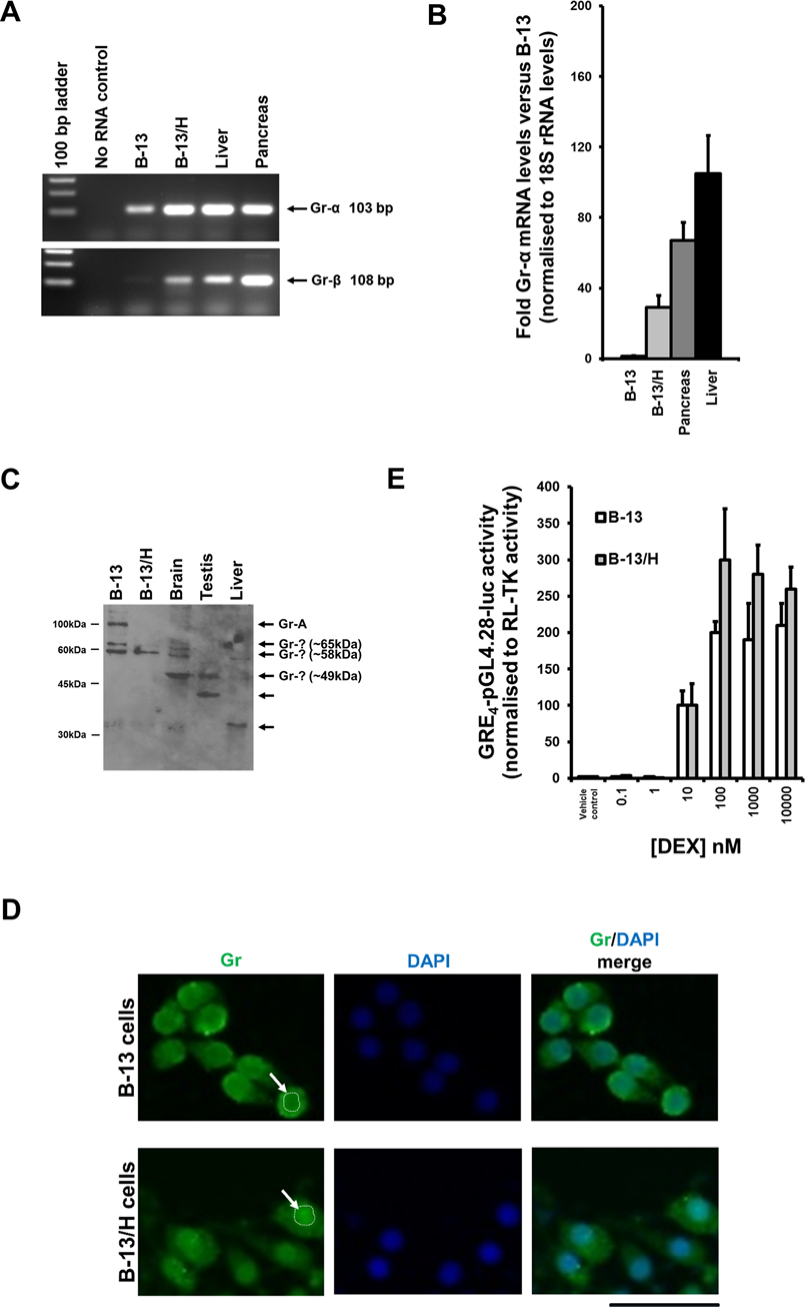
Expression and transcriptional function of Gr in B-13 and B-13/H cells. **A,** RT-PCR analysis for the expression of rat Gr mRNA transcripts (Gr-α and Gr-β) in the indicated cell and tissue samples. The Gr-β variant uses an alternate acceptor splice site at the 3' terminal exon resulting in a frame-shift and a shorter isoform with a distinct C-terminus compared to the α isoform. Data are typical of at least 4 separate B-13 and B-13/H cell cultures, after 30 cycles. **B**, Expression levels of the Gr-α mRNA transcripts relative to 18S rRNA transcript levels, and then normalised to B-13 cell. Data are the mean and standard deviation of at least 3 separate cell samples/rat tissue samples. **C**, Western blot for Gr protein, total cell protein/lane, B-13 and B-13/H 10μg/lane; rat tissue samples, 40μg/lane, typical of at least 4 separate investigations. **D**, Immunocytochemical staining for Gr (green) in B-13 and B-13/H cells with DNA stained using DAPI (blue), typical of 3 separate investigations. Arrow with circled dotted line indicates position of a cell nucleus, bar = 50μm. **E**, Gr transcriptional function as determined through transfection of GRE4-pGL4.28 and measurement of luciferase and renilla activities. Data are the mean and standard deviation of 3 separate transfections in the same experiment, typical of 3 separate experiments.

Previous work has shown that B-13 cells express the Gr mRNA and that the closely related mineralocorticoid receptor is lowly expressed and not involved in the differentiating effects of glucocorticoids in B-13 cells [12]. Fig 2A suggests that B-13 cells expressed primarily the Gr-α isoform transcript (with only low levels of the truncated variant Gr-β mRNA detectable), the orthologue of which has been shown to function in a dominant-negative fashion in man [15]. Fig 2A also suggests that the Gr-α mRNA transcript increases in abundance after conversion to B-13/H cells and this was confirmed by quantitative RT-PCR analysis (Fig 2B). However, Western blotting of cell extracts and comparison with several tissues—including liver—suggests that the Gr protein was expressed at relatively high levels in both B-13 and B-13/H cells. It has recently become clear that the human Gr-α gene produces a variety of N-terminally truncated Gr-α proteins with unique transcriptional targets and that their presence in cells is not a result of artefact proteolysis, but generated through altered translational start sites from a single mRNA species [16]. A similar phenomenon is reported to occur in rat and mouse [16]. Since the Gr gene is highly conserved, the rat and human Gr-α amino acid sequence was aligned and used to predict truncated rat Gr-α proteins and calculate their theoretical molecular weights (S2 Fig). This analysis predicted D isoforms in rat of around 50 kDa (in contrast to the full length Gr-α-A protein of 87.4 kDa). Fig 2C suggests that the full length Gr-α-A and Gr-α-D forms were present in B-13 cells and only the Gr-α-D form in B-13/H. The Gr-α-D form in human cells was reported to be localised predominantly to the nucleus and to be transcriptionally active [16]. Immunocytochemical staining in B-13 and B-13/H cells indicated that there was increased localization of Gr-α in the nucleus of B-13/H cells whereas there was no marked difference in B-13 cells (Fig 2D). Transfecting both cell phenotypes with a Gr-responsive reporter gene construct (pGRE4-pGL4.28) indicated that the Gr in B-13 cells was transcriptionally-active in a dose-dependent fashion at concentrations above approx. 1-10nM DEX. Interestingly, without the addition of DEX, the Gr in both cell phenotypes was transcriptionally-inactive (Fig 2E).

The Gr-α was therefore expressed at relatively high levels in B-13 and B-13/H cells. Activation with ligand resulted in increased Gr-α mRNA expression and increased translation of an N-terminally truncated variant transcriptionally functional protein that predominantly localized to the nucleus in B-13/H cells.

### A short 6 hour exposure to glucocorticoid is sufficient to initiate an irreversible conversion of B-13 cells to B-13/H cells

We have previously noted that the process of B-13 cell trans-differentiation into B-13/H cells appeared to be irreversible, in that removal of glucocorticoid from differentiating cells neither halts the process nor leads to a reversion back to the B-13 phenotype (unpublished observations). We therefore hypothesized that glucocorticoid exposure in B-13 cells initiates an epigenetic change(s) that accounts for the inability of B-13/H cells to revert back to B-13 cells (and which may also be critical for their near quantitative functional trans-differentiation into hepatocytes). To test this hypothesis, B-13 cells were exposed to a pulse of glucocorticoid for just 6 hours prior to extensive washing and culture in normal medium routinely used to expand the B-13 cells (i.e. without supplementation with DEX, and which does not lead to trans-differentiation into B-13/H cells).

**Fig 3:**
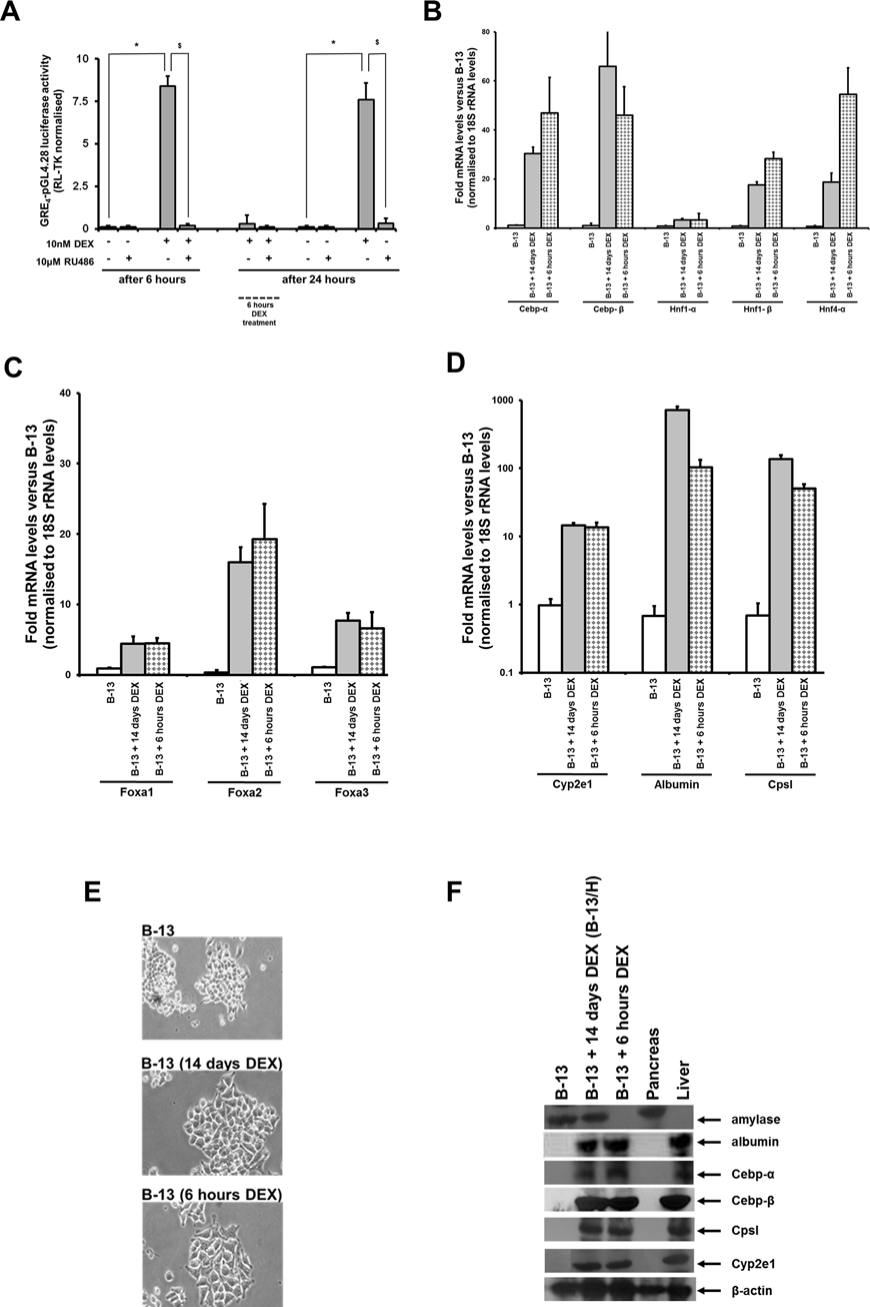
Limiting B-13 cell exposure to glucocorticoid for 6 hours results in a near quantitatively identical trans-differentiation to the B-13/H phenotype. **A**, Gr transcriptional activity in B-13 cells after the indicated period of time of treatment with DEX and/or RU486. “6 hours DEX treatment” refers to cells pulse treated with DEX for 6 hours prior to washing as outlined in methods section. Cells were transfected as outlined in methods section with GRE4-pGL4.28 and RL-TK prior to measurement of luciferase and renilla activities. Data are the mean and standard deviation of 3 separate transfections in the same experiment, typical of 3 separate experiments. *Significantly different (two tailed) normalised luciferase reporter gene activity versus control vehicle treated cells; $significantly different (two tailed) normalised luciferase reporter gene activity versus DEX only treated cells, P > 0.05. **B–D**, Expression levels of the indicated 18S rRNA normalised transcript relative to the levels present in B-13 cells (open bars) 14 days after continuous DEX treatment (grey bars) or limited 6 hours DEX treatment (hashed bars), data are the mean and standard deviation of at least 3 separate cell samples. **E**, Photomicrographs of B-13 cells 14 days after continuous DEX treatment or limited 6 hours DEX treatment. **F**, Western blot for the indicated protein in B-13 cells 14 days after continuous DEX treatment or limited 6 hours DEX treatment.

Fig 3 demonstrates that a short pulse exposure to glucocorticoid resulted in an activation of the transcriptional function of the Gr within the time frame of the pulse exposure but also confirmed that the washing procedure fully reversed Gr transcriptional activity by 24 hours (Fig 3A). Despite only a pulse of Gr activation, Fig 3B demonstrates that there was a similar induction of liver-specific/enriched transcription factors (Fig 3B and 3C) and expression of liver markers (Fig 3D) to levels close to those seen in B-13/H cells (i.e. after constant DEX exposure) after 10–14 days of culture, as determined by qRT-PCR. Furthermore, B-13 cells exposed to 6 hours of glucocorticoid formed morphologically similar cells to B-13/H cells at 10–14 days (Fig 3E), and expression of liver-specific proteins were, as seen with B-13/H cells, similar to the levels seen in intact liver (Fig 3F).

Immunocytochemistry confirmed that there was a widespread loss of amylase expression and an increase in Cepb-α and albumin expression after both DEX treatment regimens (S3 Fig). The comparable quantitative expression of mRNAs in B-13 cells treated with DEX for 6 hours only was not therefore due to a small population of cells expressing relatively high levels of these transcripts and offsetting a widespread lack of responsiveness in the majority of cells (remaining as proliferative B-13 cells).

In contrast to effectively blocking on-going activation of the Gr through washing out DEX, addition of the Gr antagonist RU486 after a period of Gr activation by DEX markedly inhibited the trans-differentiation to B-13/H cells (S4 Fig). This outcome suggests that the activation of the Gr and transcriptional effect become functionally fixed in B-13 cells when DEX is washed out, but that this effect may be blocked if the Gr transcriptional function is antagonized by subsequent exposure to a Gr antagonist.

These data demonstrate that a relatively short (6 hours) pulse exposure to glucocorticoid was sufficient to transiently activate the Gr and irreversibly drive B-13 cell trans-differentiation to B-13/H cells to a quantitatively similar hepatic level 10–14 days later, in the absence of Gr antagonist. This response suggests that the effects triggered around the time period of pulse exposure (less than 24 hours) is sufficient to direct B-13 cells toward an hepatic B-13/H phenotype.

### Inhibition of glucocorticoid-initiated epigenetic changes in B-13 cells blocks trans-differentiation into B-13/H cells

**Fig 4:**
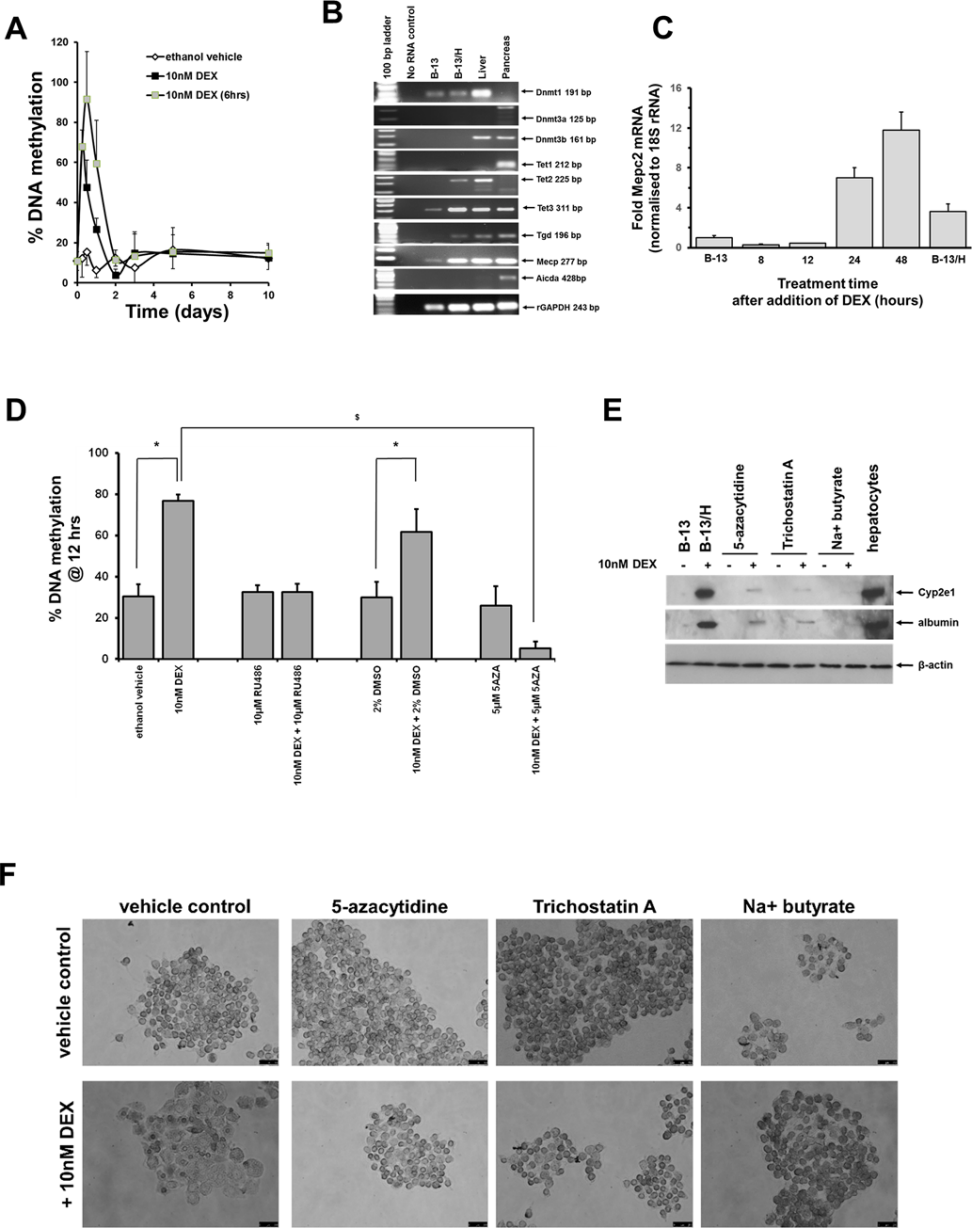
Glucocorticoid treatment results in a pulse of DNA methylation that is dependent on the Gr and is essential for B-13 cell trans-differentiation to B-13/H cells. **A**, genomic DNA methylation in B-13 cells treated as indicated [DEX (6 hrs), B-13 cells treated with a pulse 6 hour treatment]. **B**, RT-PCR analysis for the expression of the indicated transcript in the indicated cell and tissue samples. **C**, qRT-PCR for Mecp2 mRNA in the indicated time point, expressed relative to the levels present in B-13 cells. Data are the mean and standard deviation of 3 determinations from the same experiment, typical of 3 separate experiments. **D**, genomic DNA methylation in B-13 cells at the peak of methylation after DEX treatment (12 hours) with treatments as indicated. Data are the mean and standard deviation of 3 determinations from the same experiment, typical of 3 separate experiments. ^*^Significantly different (two tailed) DNA methylation versus control vehicle treated cells; ^$^significantly different (two tailed) DNA methylation versus DEX only treated cells, P > 0.05. **E**, Western blot for the indicated proteins in B-13 cells treated for 14 days as indicated, results typical of at least 3 separate determinations. **F**, Photomicrographs of B-13 cells 7 days after continuous DEX treatment with or without the indicated epigenetic inhibitors or vehicle control (cells were pre-treated with these inhibitors 2 hours before first exposure to DEX). Medium was changed as outlined in methods section with DEX and epigenetic inhibitor treatments renewed at each media change (results typical of at least 8 separate experiments.

To test the hypothesis that an epigenetic change(s) is critical in the trans-differentiation of B-13 cells to B-13/H cells, the methylation of DNA was examined at various time points after the addition of DEX. Fig 4A demonstrates that genomic DNA was methylated within hours of exposure to DEX, peaking around 12 hours, followed by de-methylation and apparent stabilization throughout subsequent morphological changes to B-13/H cells. Pulse exposure to DEX resulted in a similar peak in DNA methylation (Fig 4A). A screen for the expression of a variety of DNA methylation transcripts indicated that a restricted number were expressed in B-13/H cells compared to rat liver, and that expression of all those examined were relatively low in B-13 cells (Fig 4B). To test for a potential functional effect of DNA methylation in B-13 cells exposed to DEX, the levels of the Mecp2 mRNA were examined by qRT-PCR, since the protein product of this gene binds to methylated DNA as part of the methylation-associated mechanism of gene repression or induction [17]. Fig 4C demonstrates that there was an induction of Mecp2 mRNA transcripts after the pulse in DNA methylation that was maximal (approx. 12-fold) 48 hours after exposure to DEX, suggesting that the pulse in DNA methylation may be functional and have a significant functional role in B-13 cell differentiation. To test this hypothesis, B-13 cells were pre-treated with the DNA methylation inhibitor 5-azacytidine (5AZA) [18]. The effect of high concentrations of DMSO (2% [v/v])–which promotes the hydroxylation of methylated DNA [19]–was also examined. Fig 4D demonstrates that the Gr antagonist RU486, which is known to block glucocorticoid-dependent B-13 trans-differentiation to B-13/H cells [1,12], inhibited DEX-dependent DNA methylation, confirming that the pulse in DNA methylation is Gr-dependent. Fig 4D also demonstrates that 5AZA significantly inhibited DEX-induced DNA methylation, whereas DMSO had minimal effect. Both 5AZA and DMSO (S5 Fig)–in addition to inhibitors of histone modifications trichostatin A and sodium butyrate [20,21] markedly inhibited DEX-induced B-13 trans-differentiation to B-13/H cells based on almost complete inhibition of hepatic Cyp2e1 and albumin protein expression (Fig 4E) and inhibition of morphological changes (Fig 4F).

**Fig 5:**
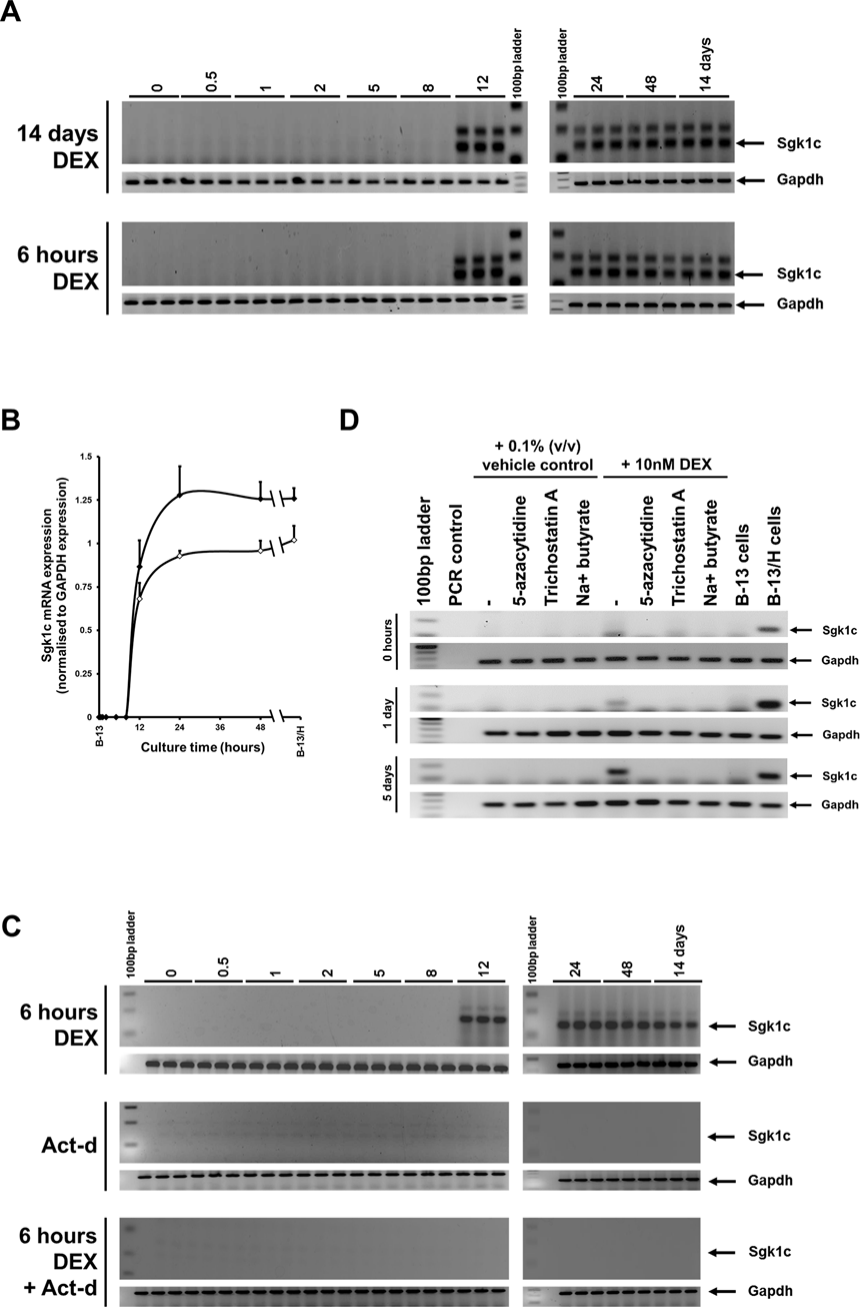
DEX-induced Sgk1c mRNA expression in B-13 cells is transcriptionally induced from constitutively low levels in B-13 cells and induction is imprinted by pulse DEX exposure. **A**, Gel electrophoresis of RT-PCR products designed to amplify a region specific to the rat Sgk1c mRNA transcript (143 bp as indicated). The identity of the high molecular weight band is unknown and was not always evident between different experiments. B-13 cells were treated in triplicate with 10nM DEX continuously for 14 days or for a 6 hour pulse as indicated and RNA isolated and Sgk1C and Gapdh mRNA transcript levels examined by RT-PCR at the indicated times. Each separate experiment is shown. B-13 cells treated with 0.1% (v/v) ethanol (i.e. vehicle control) had minimal effect on all transcripts were as for time 0 (not included). After 3–4 days, B-13 cells require sub-culturing since they continue to proliferate. Therefore, they are not appropriate controls for B-13 cells treated with DEX after this time since DEX treatment results in a cessation of proliferation and sub-culture is not required and also leads to significant cell loss. **B**, semi-quantitative image analysis of Sgk1c band intensity, mean and standard deviation of the three separate experiments shown in A. **C**, Effect of actinomycin d (0.3μg/ml) on the induction of Sgk1c mRNA transcript in response to 6 hours exposure to 10nM DEX. After 6 hours exposure to the indicated treatments, cells were washed and cultured in standard media with DEX or actinomycin d. Each separate experiment is shown. **D**, Effect of the indicated epigenetic inhibitor on the induction of Sgk1c mRNA transcript in response to continuous exposure to 10nM DEX in B-13 cells, time after treatment initiation as indicated on left of panels. Results typical of 3 separate experiments.

Previous experiments with B-13 cells have identified N-terminal Sgk1 variant proteins as pivotal to the mechanism(s) associated with pancreatic–hepatic differentiation. Notably, ectopic expression of 2 human variants–C (also termed variant 3) and F–in B-13 cells was sufficient to promote B-13 trans-differentiation to B-13/H cells in the absence of glucocorticoid. Expression of other human SGK1 isoforms, including the wild type A (or variant 1) had no effect on B-13 cell trans-differentiation to B-13/H cells [12]. Since a 6 hour pulse exposure to DEX was sufficient to initiate B-13 cell trans-differentiation to B-13/H cells, a comparison of the effects of constant DEX and a 6 hour pulse of DEX treatment on Sgk1c mRNA expression was examined. Because the Sgk1c mRNA transcript contains only a short 5’ nucleotide sequence specific to the transcript, a primer pair suitable for use in qRT-PCR could not be designed that specifically amplified a region of this transcript. Accordingly, a semi-quantitative RT-PCR assay was developed and transcript levels quantified by image analysis of the predicted PCR product band in ethidium bromide stained gels. Fig 5A and 5B demonstrates that a 6 hour exposure to DEX was sufficient to result in a near-similar robust increase in Sgk1c mRNA expression from undetectable levels in B-13 cells, when compared to cells exposed throughout with DEX. Fig 5C further indicates that the mechanism of induction of Sgk1c mRNA was wholly transcriptional since the RNA polymerase inhibitor actinomycin d [22] blocked Sgk1c mRNA expression in response to hours DEX. Note, that a variant orthologous to the F variant has not been identified in rat. Addition of DNA methylation inhibitor 5-azacytidine or the inhibitors of histone modifications—trichostatin A and sodium butyrate–blocked the induction of Sgk1c mRNA transcript expression by DEX (Fig 5D), suggesting that the constitutive expression of this transcript after DEX exposure is dependent on DEX-dependent epigenetic alterations in B-13 cells.

These data therefore show that exposing B-13 cells to DEX results in a Gr-dependent pulse in DNA methylation and likely other epigenetic changes such as histone modifications and to the constitutive expression of Sgk1c, even after only transient DEX exposure.

## Discussion

The B-13 cell is a unique cell line in that its differentiation into hepatocyte-like B-13/H cells results in a relatively quantitatively comparative cell to its *in vivo* hepatocyte equivalent. To date, the only factors known to trigger this response are glucocorticoids *via* the Gr nuclear receptor. The mechanisms involved in the formation of B-13/H cells involve an induction of selected Sgk1 isoforms which cross talk into the Wnt signalling pathway leading to a transient repression in Wnt activity and induction of Cebp-β [5, 12, 1]. Similar observations have been observed *in vivo* under pathological conditions, suggesting that the B-13 cell over-responds to physiologically relevant glucocorticoid in an analogous manner to that observed in normal pancreatic acinar cells in response to injury/stress [23, 24] (for a recent review see also [3]) and to high pathological levels of systemic glucocorticoid [5].

This paper confirms for the first time, that glucocorticoid exposure in B-13 cells resulted in a B-13/H phenotype that is remarkably similar to an hepatocyte phenotype as judged on the quantitative levels of expression of liver-specific, liver-enriched transcription factor and hepatocyte defining mRNAs and that a pulse exposure to glucocorticoid was sufficient to irreversibly convert B-13 cells to B-13/H cells. Exposure to glucocorticoid led to a pulse in DNA methylation and conversion to B-13/H cells that were blocked by the DNA methylation inhibitor 5AZA, suggesting that DNA methylation is essential for B-13 cell trans-differentiation to B-13/H cells and constitutes a component of the mechanism that prevents reversion from B-13/H to B-13 cell. High concentrations of DMSO also blocked B-13 cell trans-differentiation to B-13/H, suggesting that hydroxylation of methylated DNA inhibits the formation of an hepatic phenotype. Histone deacetylase inhibitors also blocked B-13 cell trans-differentiation to B-13/H, indicating that histone modification is also a component of this mechanism.

Previous work has demonstrated that a major induced transcript in B-13 cells exposed to glucocorticoid is that encoding Sgk1 [4]. More recent evidence strongly implicates variant Sgk1 transcripts as pivotal in the process of pancreatic–hepatic differentiation. In transgenic mice with high levels of circulating endogenous glucocorticoid, pancreata expressing a variety of liver markers also expressed detectable levels of the murine orthologue of Sgk1c, whereas wild type mice expressed neither liver markers nor Sgk1c mRNA [25, 12]. In pancreata from patients undergoing pancreatectomies, the C and F isoform mRNAs of SGK1 where not detectable. In contrast, the pancreas from a patient exposed long term to systemic glucocorticoid treatment and whose pancreas expressed high (liver levels) of a range of hepatic proteins, expressed readily detectable levels of the C and F mRNA isoforms [6]. In human acinar cells in culture that expressed hepatic markers in response to high concentrations of DEX, both C and F isoform mRNAs were also induced, whereas in acinar cells which did not respond to DEX, there was no induction of these isoforms [6]. Since Sgk1c mRNA expression remains constitutively expressed in B-13 cells after transcriptional induction by transient exposure to DEX, it is likely that epigenetic alterations are a major mechanism responsible for this apparent irreversible response. Future work is currently being directed to identifying the precise epigenetic changes controlling Sgk1c expression in B-13 cells since it may be applied to stem cells and potentiate differentiated phenotypes *in vitro*.

The advent of the REACH regulations in the EU means that a large number of chemicals will have to be assessed for their safety, a requirement that precludes the widespread use of animals in toxicity testing on both financial and ethical Grounds. A major goal in Toxicology is therefore to find computational and *in vitro* methods to screen chemicals for potential toxicities. Given the target organ toxicity paradigm, near equivalent functional comparability of *in vitro* cells to *in vivo* is important if *in vitro* assessments are to be predictive of human susceptibility when dealing with the general population and exposures. This is particularly relevant for the liver, where high levels of chemical metabolising enzymes are a major determinant in hepatotoxicity [26]. In view of the shortages, costs and variability in obtaining human hepatocytes, stem cell-derived hepatocytes are a realistic alternative for toxicity screening. However, stem and progenitor cells currently fail to generate fully differentiated functional mature cells *in vitro* despite their intrinsic capacity to do so *in vivo* [11]. An understanding of the mechanism(s) involved in the trans-differentiation to B-13/H cells may inform on the approaches required to generate a human equivalent.

## Supporting Information

S1 Fig Derivation of a reporter construct conferring glucocorticoid receptor (Gr) responsiveness. **A,** alignment of several Gr response elements and derivation of a consensus glucocorticoid response element (GRE) sequence. Alignment of the sequences of the GREs from the tyrosine amimontransferase gene (TAT); the tryptophan oxygenase gene (TO); the human metallothionein gene, hMT; the murine sarcoma virus (MSV), the human growth hormone gene (hGH) and the mouse mammary tumour virus (MMTV) as reported by Jantzen et al., 1987. **B**, sequence of 4 GREs (GRE4-) cloned into the pGL4.28 luciferase reporter construct as outlined in methods section. **C**, GRE4- sequence conferred responsiveness to DEX. B-13 cells were seeded into 6 well plates 24 hours prior to transfection and cultured in standard culture media. Cells were transfected with 2 µg/well pGL4.28 or GRE4–pGL4.28 reporter plasmid and 0.2 µg/well renilla luciferase (RL-TK) using Lipofectamine® transfection reagent, as outlined by the manufacturer, left for 48 hours prior to treatment with either control vehicle (0.1% [v/v] ethanol) or DEX as indicated. Luciferase activities were analysed using a Dual Glo® luciferase assay system (Promega, Southampton, UK) and a luminometer. Luciferase activities were normalised to renilla activities, to control for any variations in transfection efficiencies between wells. Data are the mean and standard deviation of 3 separate experiments, typical of 3 separate experiments. *Significantly different (two tailed) normalised luciferase reporter gene activity versus control vehicle treated cells, P > 0.05.
(TIF)

S2 Fig Alignment of amino acid sequences of rat and human GRα proteins. CLUSTAL O (1.2.0) multiple sequence alignment was used to align the sequences. Molecular weights were of rat GRα isoforms was calculated using software available at http://web.expasy.org/compute_pi/.
(PDF)

S3 Fig Immunocytochemical staining of B-13 and B-13/H cells. Immunocytochemical staining for the indicated antigen in B-13 cells 14 days after either continuous DEX treatment or limited 6 hours DEX treatment after subsequent culture to 14 days, No 1° Ab CONTROL MERGE, DAPI and FITC fluorescence merge after identical incubations with the exception of the primary antibody. Results typical of at least 3 separate experiments.
(TIF)

S4 Fig Effect of subsequent exposure to Gr antagonism on pulsed DEX-induced B-13 cell trans-differentiation to B-13/H cells. Western blot for the indicated proteins in B-13 cells treated with DEX or 0.1% ethanol vehicle control (-) for 2 days (^*^or continuously with DEX for 14 days to generate B-13/H cells) followed by washing and treatment with RU486 or 0.1% ethanol vehicle control (-) for the subsequent 5 days. Cells were analyzed at the time points indicated. Results typical of at least 3 separate determinations.
(TIF)

S5 Fig Effect of DMSO on DEX-induced B-13 cell trans-differentiation to B-13/H cells. Western blot for the indicated proteins in B-13 cells treated for 14 days as indicated, results typical of at least 3 separate determinations.
(TIF)
