# Supplementary figures and images for: Pancreatic B-13 Cell Trans-Differentiation to Hepatocytes Is Dependent on Epigenetic-Regulated Changes in Gene Expression
Source: PLoS One. 2016 Mar 8;11(3):e0150959. doi: 10.1371/journal.pone.0150959 (PMC4782989; doi:10.1371/journal.pone.0150959)

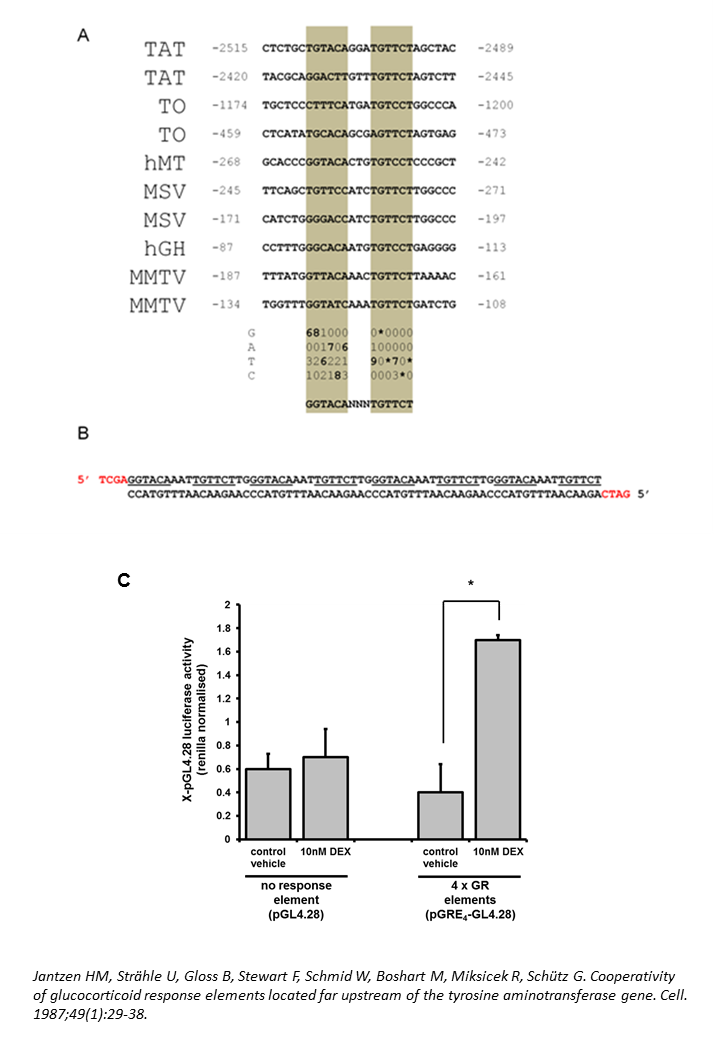

Supplement: S1 Fig — A, alignment of several Gr response elements and derivation of a consensus glucocorticoid response element (GRE) sequence. Alignment of the sequences of the GREs from the tyrosine amimontransferase gene (TAT); the tryptophan oxygenase gene (TO); the human metallothionein gene, hMT; the murine sarcoma virus (MSV), the human growth hormone gene (hGH) and the mouse mammary tumour virus (MMTV) as reported by Jantzen et al., 1987. B, sequence of 4 GREs (GRE4-) cloned into the pGL4.28 luciferase reporter construct as outlined in methods section. C, GRE4- sequence conferred responsiveness to DEX. B-13 cells were seeded into 6 well plates 24 hours prior to transfection and cultured in standard culture media. Cells were transfected with 2 µg/well pGL4.28 or GRE4–pGL4.28 reporter plasmid and 0.2 µg/well renilla luciferase (RL-TK) using Lipofectamine® transfection reagent, as outlined by the manufacturer, left for 48 hours prior to treatment with either control vehicle (0.1% [v/v] ethanol) or DEX as indicated. Luciferase activities were analysed using a Dual Glo® luciferase assay system (Promega, Southampton, UK) and a luminometer. Luciferase activities were normalised to renilla activities, to control for any variations in transfection efficiencies between wells. Data are the mean and standard deviation of 3 separate experiments, typical of 3 separate experiments. *Significantly different (two tailed) normalised luciferase reporter gene activity versus control vehicle treated cells, P > 0.05. (TIF) [file pone.0150959.s001.TIF]

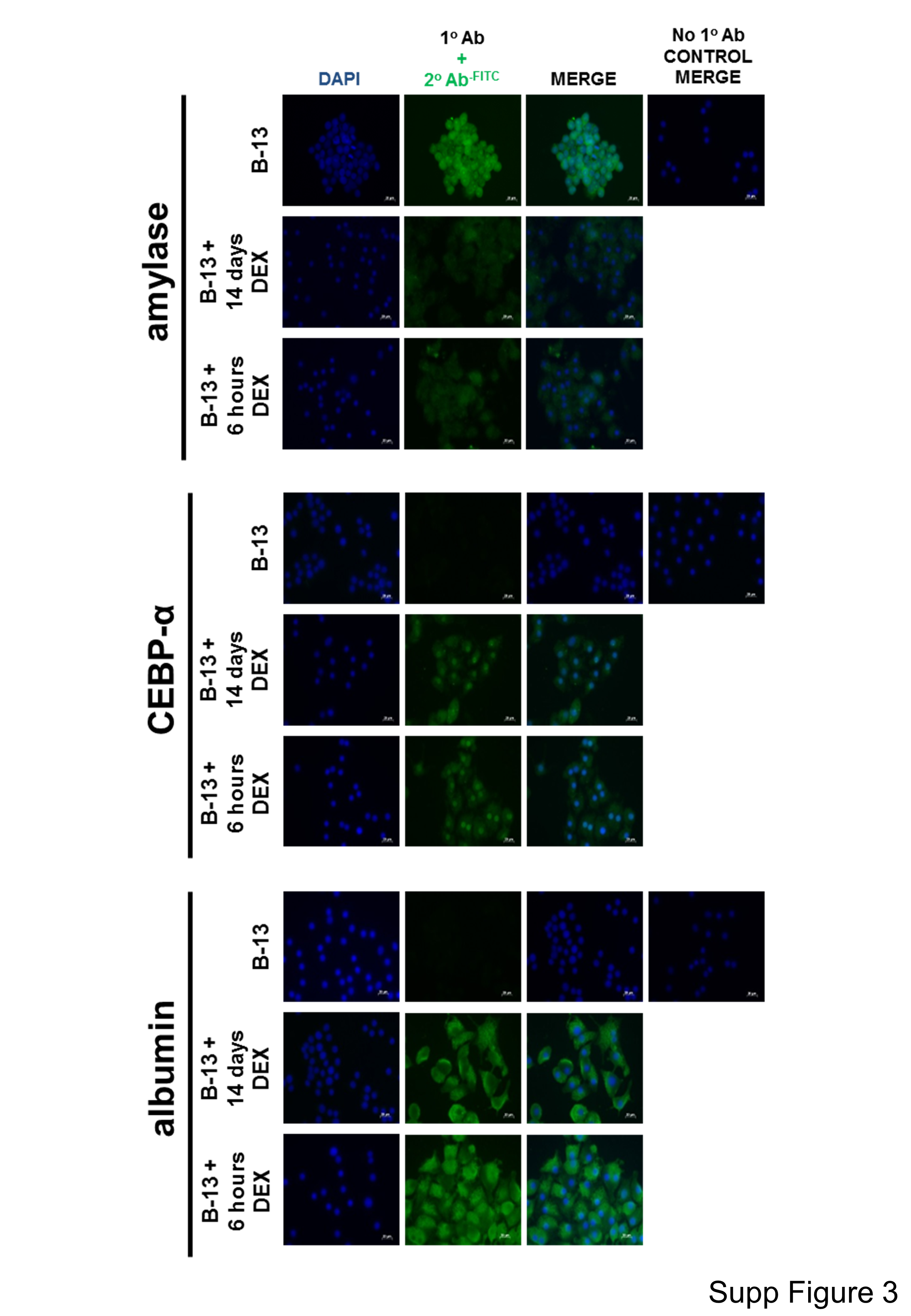

Supplement: S3 Fig — Immunocytochemical staining for the indicated antigen in B-13 cells 14 days after either continuous DEX treatment or limited 6 hours DEX treatment after subsequent culture to 14 days, No 1° Ab CONTROL MERGE, DAPI and FITC fluorescence merge after identical incubations with the exception of the primary antibody. Results typical of at least 3 separate experiments. (TIF) [file pone.0150959.s003.TIF]

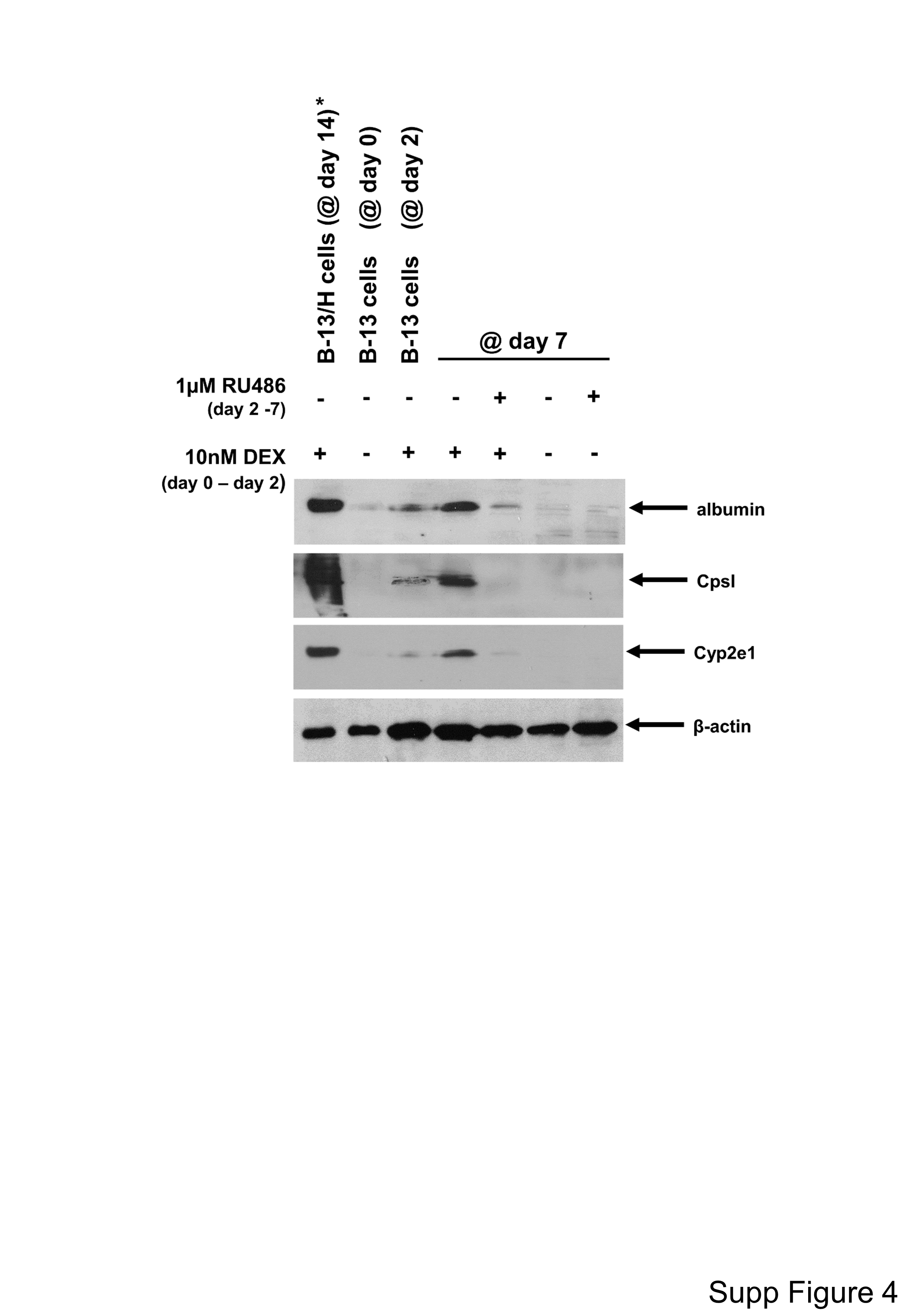

Supplement: S4 Fig — Western blot for the indicated proteins in B-13 cells treated with DEX or 0.1% ethanol vehicle control (-) for 2 days (*or continuously with DEX for 14 days to generate B-13/H cells) followed by washing and treatment with RU486 or 0.1% ethanol vehicle control (-) for the subsequent 5 days. Cells were analyzed at the time points indicated. Results typical of at least 3 separate determinations. (TIF) [file pone.0150959.s004.TIF]

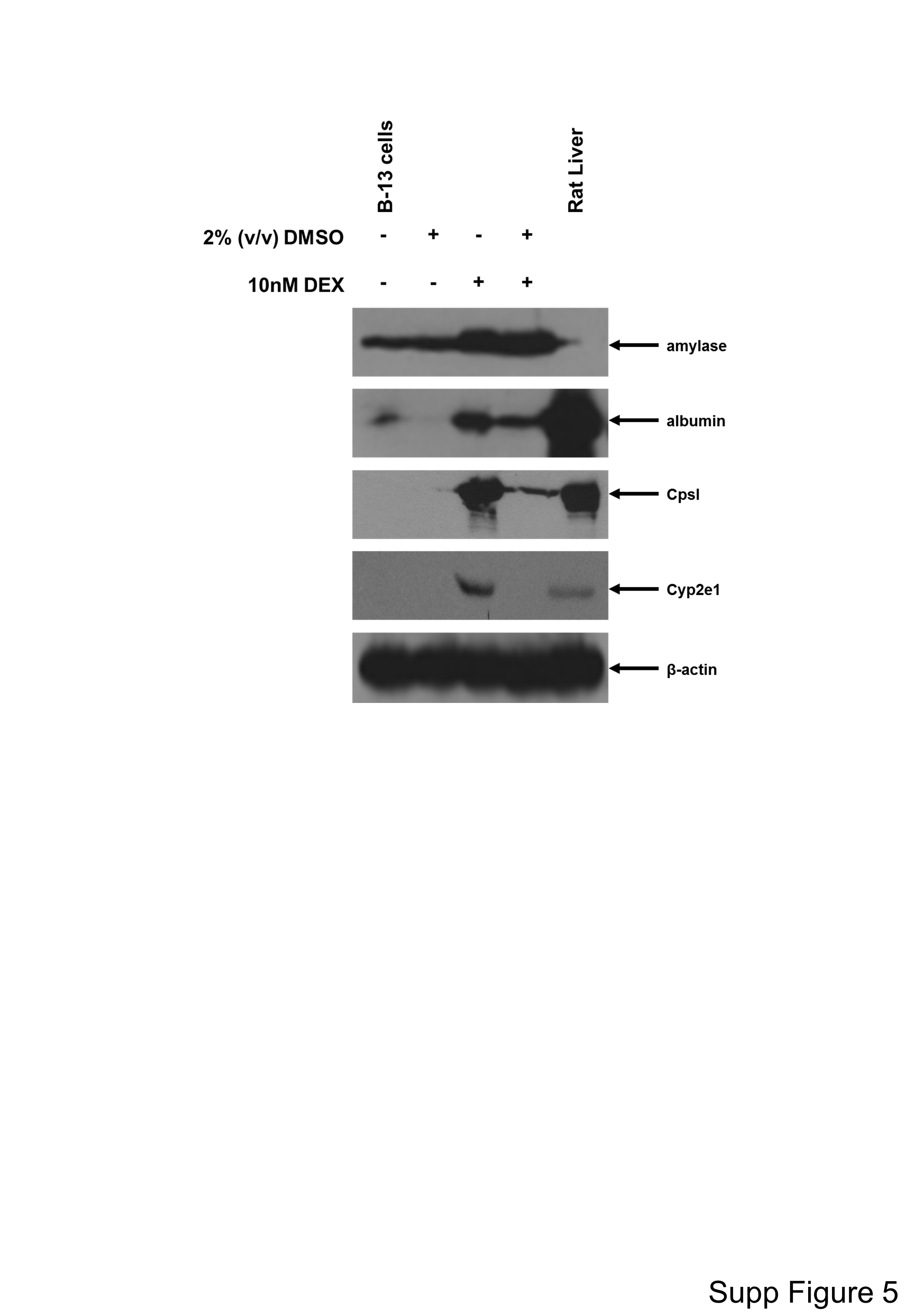

Supplement: S5 Fig — Western blot for the indicated proteins in B-13 cells treated for 14 days as indicated, results typical of at least 3 separate determinations. (TIF) [file pone.0150959.s005.TIF]
