# Supplementary material for: Pancreatic B-13 Cell Trans-Differentiation to Hepatocytes Is Dependent on Epigenetic-Regulated Changes in Gene Expression
Source: PLoS One. 2016 Mar 8;11(3):e0150959. doi: 10.1371/journal.pone.0150959 (PMC4782989; doi:10.1371/journal.pone.0150959)
Supplement: S2 Fig — CLUSTAL O (1.2.0) multiple sequence alignment was used to align the sequences. Molecular weights were of rat GRα isoforms was calculated using software available at http://web.expasy.org/compute_pi/. (PDF) [file pone.0150959.s002.pdf]

Supplemental Figure 2. Alignment of amino acid sequences of rat and human GRα proteins using CLUSTAL O(1.2.0) multiple sequence alignment and predicted size of rat GRα isoforms (using software available at http://web.expasy.org/compute_pi/)

```
         GRα-A→→                    GRα-B→→

rGR      MDSKESLAPPGRDEVPGSLLGQGRGSVMDFYKSLRGGATVKVSASSPSVAAASQADSKQQ

hGR      MDSKESL-TPGREENPSSVLAQERGDVMDFYKTLRGGATVKVSASSPSLAVASQSDSKQR

         *******  ***:* *.*:*.* **.******:***************:*.***:****:

                                                  GRα-C1

                                                       GRα-C2→→

                                                              GRα-C3→→

rGR      RILLDFSKGSTSNVQQRQQQQQQQQQQQQQQQQQQQPDLSKAVSLSMGLYMGETETKVMGN

hGR      RLLVDFPKGSVSNA-------------------QQPDLSKAVSLSMGLYMGETETKVMGN

         *:*:** ***.**.                   ***************************


rGR      DLGYPQQGQLGLSSGETDFRLLEESIANLNRSTSVPENPKSSTSATGCATPTEKEFPKTH

hGR      DLGFPQQGQISLSSGETDLKLLEESIANLNRSTSVPENPKSSASTAVSAAPTEKEFPKTH

         ***:*****:.*******::**********************:*:: .*:**********


rGR      SDASSEQQNRKSQTGTNGGSVKLYPTDQSTFDLLKDLEFSAGSPGKDTNESPWRSDLLID

hGR      SDVSSEQQHLKGQTGTNGGNVKLYTTDQSTFDILQDLEFSSGSPGKETNESPWRSDLLID

         **.*****. *.*******.**** *******:*:*****:*****:*************


rGR      EN-LLSPLAGEDDPFLLEGDTNEDCKPLILPDTKPKIKDTGDTILSSPSSVALPQVKTEK

hGR      ENCLLSPLAGEDDSFLLEGNSNEDCKPLILPDTKPKIKDNGDLVLSSPSNVTLPQVKTEK

         ** ********** *****::******************.** :*****.*:********

                                        GRα-D1→→

                                                       GRα-D2→→

                                                            GRα-D3→→

rGR      DDFIELCTPGVIKQEKLGPVYCQASFSGTNIIGNKMSAISVHGVSTSGGQMYHYDMNTAS

hGR      EDFIELCTPGVIKQEKLGTVYCQASFPGANIIGNKMSAISVHGVSTSGGQMYHYDMNTAS

         :***************** ******* *:*******************************


rGR      LSQQQDQKPVFNVIPPIPVGSENWNRCQGSGEDSLTSLGALNFPGRSVFSNGYSSPGMRP

hGR      LSQQQDQKPIFNVIPPIPVGSENWNRCQGSGDDNLTSLGTLNFPGRTVFSNGYSSPSMRP
```

```
        *********:*********************:*.*****:******:*********.***

rGR     DVSSPPSSSS-AATGPPPKLCLVCSDEASGCHYGVLTCGSCKVFFKRAVEGQHNYLCAGR
hGR     DVSSPPSSSSTATTGPPPKLCLVCSDEASGCHYGVLTCGSCKVFFKRAVEGQHNYLCAGR
        ********** *:***********************************************

rGR     NDCIIDKIRRKNCPACRYRKCLQAGMNLEARKTKKKIKGIQQATAGVSQDTSENP-NKTI
hGR     NDCIIDKIRRKNCPACRYRKCLQAGMNLEARKTKKKIKGIQQATTGVSQETSENPGNKTI
        *******************************************:*****:***** ****

rGR     VPAALPQLTPTLVSLLEVIEPEVLYAGYDSSVPDSAWRIMTTLNMLGGRQVIAAVKWAKA
hGR     VPATLPQLTPTLVSLLEVIEPEVLYAGYDSSVPDSTWRIMTTLNMLGGRQVIAAVKWAKA
        ***:*******************************:************************

rGR     IPGFRNLHLDDQMTLLQYSWMFLMAFALGWRSYRQSSGNLLCFAPDLIINEQRMSLPCMY
hGR     IPGFRNLHLDDQMTLLQYSWMFLMAFALGWRSYRQSSANLLCFAPDLIINEQRMTLPCMY
        *************************************.****************:*****

rGR     DQCKHMLFVSSELQRLQVSYEEYLCMKTLLLLSSVPKEGLKSQELFDEIRMTYIKELGKA
hGR     DQCKHMLYVSSELHRLQVSYEEYLCMKTLLLLSSVPKDGLKSQELFDEIRMTYIKELGKA
        *******:*****:***********************:**********************

rGR     IVKREGNSSQNWQRFYQLTKLLDSMHEVVENLLTYCFQTFLDKTMSIEFPEMLAEIITNQ
hGR     IVKREGNSSQNWQRFYQLTKLLDSMHEVVENLLNYCFQTFLDKTMSIEFPEMLAEIITNQ
        *********************************.**************************

rGR     IPKYSNGNIKKLLFHQK
hGR     IPKYSNGNIKKLLFHQK
        *****************
```

**Protein**	**Size (kDa)**
GRα-A	87.4
GRα-B	84.7
GRα-C1	76.1
GRα-C2	75.6
GRα-C3	74.8
GRα-D1	51.5
GRα-D2	50.1
GRα-D3	49.4
